# Dynamics of Serum Inflammatory Markers and Adipokines in Patients: Implications for Monitoring Abnormal Body Weight: Preliminary Research

**DOI:** 10.3390/metabo14050260

**Published:** 2024-05-01

**Authors:** Malwina Zimowska, Marta Rolbiecka, Klaudia Antoniak-Pietrynczak, Marta Jaskulak, Katarzyna Zorena

**Affiliations:** Department of Immunobiology and Environment Microbiology, Faculty of Health Sciences, Medical University of Gdansk, Debinki 7, 80-211 Gdańnsk, Poland; malwinazimowska@gumed.edu.pl (M.Z.); m.rolbiecka.976@studms.ug.edu.pl (M.R.); klaudia.antoniak@gumed.edu.pl (K.A.-P.); katarzyna.zorena@gumed.edu.pl (K.Z.)

**Keywords:** patients, obesity, overweight, IL-6, ZAG, nesfatin-1, adiponectin, leptin, hsCRP, lipid profile, BMI

## Abstract

This study aimed to investigate the influence of abnormal body weight on inflammatory markers and adipokine levels across varied body mass index (BMI) categories. The cohort included 46 participants categorized into normal BMI (group I; *n* = 19), overweight (group II; *n* = 14), and obesity (group III; *n* = 13). Inflammatory markers (hsCRP and IL-6) and adipokines (Adiponectin, Leptin, Nesfatin-1, and Zinc-α2-glycoprotein) were assessed to discern effective indicators of inflammation in individuals with abnormal body weight. Additionally, the full lipid profile was also assessed (total cholesterol, triglycerides, LDL-C, HDL-C). The results indicated significant biochemical changes, particularly in IL-6 and Leptin levels, in participants with a BMI over 25. The levels of ZAG protein were negatively correlated with the HDL-C and LDC-L levels with statistical significance (Pearson: −0.57, *p* = 0.001, and Pearson: −0.41, *p* = 0.029, for HDL-C and LDL-C, respectively), suggesting that the level of ZAG is also inversely proportional to the amount of cholesterol. Statistical analyses revealed decreased Zinc-α2-glycoprotein (ZAG) levels and increased Adiponectin, Leptin, and IL-6 levels in individuals with abnormal body weight. Correlation analyses demonstrated a statistically significant upward trend for IL-6 (*p* = 0.0008) and Leptin (*p* = 0.00001), with a similar trend observed for hsCRP without statistical significance (*p* = 0.113). IL-6 levels in the overweight group were 158.71% higher than in the normal-weight group, while the obese group exhibited a 229.55% increase compared to the normal-weight group. No notable changes have been recorded for the levels of Nesfatin-1. Based on our results, we propose IL-6, Leptin, and ZAG as potential biomarkers for monitoring interventions and assessing patient conditions in those with abnormal BMIs. Further research with a larger patient cohort is warranted to validate these correlations in overweight and obese individuals.

## 1. Introduction

The World Health Organization defines obesity as a complex multifactor disease leading to an increased risk of many illnesses. Currently, overweight and obesity affect approximately 60% of adults in the WHO European Region [1], posing significant public health risks [2]. According to research, obesity is a multifactorial disease [2,3,4]. Genetic, hormonal and environmental factors, including unlimited access to food and reduced physical activity, contribute to the increase in obesity in both children and adults [5]. Abnormal adipocyte growth, along with an excessive mass, results in reduced blood supply and oxygen delivery to the tissues [6]. This is manifested by local inflammation and, as a consequence, may lead to many diseases, including cancer, ischemic heart disease, stroke, hypertension, type 2 diabetes, etc. [6,7,8]. Adipocytes have the ability to produce adipokines, pro-inflammatory cytokines, growth factors, and other biologically active factors. Research has shown that about one-third of the total concentration of this cytokine originates in adipose tissues, while the remainder comes from many different cells [7]. Depending on the concentration, IL-6 receptors in many brain areas affect appetite and energy intake [8]. Abnormal IL-6 levels can cause cardiovascular complications and contribute to insulin resistance or type 2 diabetes [7]. Elevated IL-6 levels correlate with hsCRP levels, fibrinogen, white blood cells, and platelets [8]. Chronic inflammation resulting from obesity lasts several days to years and manifests at the tissue level. In this case, adipose tissue causes the release of inflammatory mediators, in addition to, i.a., macrophages that transport acute-phase proteins (e.g., hsCRP) into the bloodstream [9,10].

Adipose tissue secretes various proteins, such as adipokines, whose imbalance negatively impacts adipocytes, leading to hypertrophy or inflammation. This, in turn, affects the function of several organs, including the brain, liver, and pancreas, influencing processes like appetite regulation, insulin secretion, and blood pressure [6]. In the case of obesity, the expression of adipokines from anti-inflammatory to pro-inflammatory causes changes in metabolism (in obese patients, insulin resistance appears, but the risk of cardiovascular diseases also increases) [11]. Therefore, adipokines are used as biomarkers for the early detection of metabolic diseases [7]. Leptin is a hormone that regulates food intake and affects body weight and immune responses. The level of circulating Leptin decreases due to the restriction of food intake and increases during overfeeding [12]. 

Another factor that can influence obesity is Nesfatin-1. It regulates many functions through secretion in different places (e.g., by the neurons and visceral adipose tissue) [13]. Recently, there has been an increasing interest in the effects of Nesfatin-1 on appetite levels, glucose levels (increases glucose-induced insulin secretion), and fat gain (although results vary) [14].

Another important protein in regulating body weight, glucose levels, and body fat is Zinc-α2-glycoprotein (ZAG) [15,16,17,18,19]. Initially detected in plasma, ZAG was subsequently discovered in several other organs, such as mammary glands. It has been proven that adipose tissue is responsible for the secretion of ZAG [16]. This glycoprotein has various functions in the body, including its potential role as a marker of some cancers, aiding in the diagnosis and prognosis of cachexia, though its full range of functions remains incompletely understood. Its expression is affected by androgens and progestins, while glucocorticoids are responsible for its increased levels [17]. ZAG, found in adipose tissue and adipocytes, participates in regulating body weight, glucose, and lipid metabolism. It inhibits lipogenesis and stimulates lipolysis [16]. As an adipokine, it regulates the function of adipose tissue locally [18]. 

Another adipokine is Adiponectin, secreted by fat cells [10,20]. It performs metabolic functions in the liver, by activating glucose transport and increasing insulin sensitivity, and skeletal muscles, by enhancing insulin sensitivity. Additionally, Adiponectin in adipose tissue increases glucose uptake and affects fat metabolism. It exhibits a protective effect in some inflammatory diseases by affecting inflammatory cells [20]. 

Epidemiological studies also suggest an effect on cell proliferation: reduced levels of Adiponectin are linked with an increased risk of breast or endometrial cancer (there is also a relationship between increased levels and increased risk of, for example, stomach cancer). Its expression and secretion decrease in patients with obesity; however, in males, this level is naturally lower than in females. When weight is lost, Adiponectin levels return to normal. Metabolic diseases are believed to be associated with various mutations [20]. In addition, the supply of globular Adiponectin in therapy increases its plasma level, which reduces the risk of obesity and type 2 diabetes [6].

Our previous studies have already discussed the relationship between Adiponectin, cytokines or hsCRP, and body weight in the context of the impact of manual lymphatic drainage on selected parameters in obesity [21,22,23]. This study aimed to investigate the influence of abnormal body weight on inflammatory markers and adipokine levels across varied BMI categories.

## 2. Materials and Methods

### 2.1. Data Collection

This study included 46 patients. The patients enrolled in the study according to the guidelines of the Polish Diabetes Society were divided into three groups: patients with normal body weight (as a control group; group I = 19), patients that were overweight (group II = 14), and patients with obesity (group III = 13). The degree and type of obesity were determined based on the BMI values according to the guidelines of the Polish Diabetes Society [24]. This study was approved by the Bioethics Committee for Scientific Research at the Medical University of Gdańsk (protocol code NKBBN/692/2019–2020; date of approval: 30 January 2020). Informed consent was collected from all participants.

For each patient, anthropometric measurements were taken using the same scales. Height measurements were performed with an accuracy of 0.5 cm and body weight measurements with an accuracy of 0.1 kg. The patients wore minimal clothing, were barefoot, and were commanded to stand still. The body mass index (BMI) was calculated according to the following formula: BMI = body weight (kg)/height (m)^2^. The Tanita SC-240 foot-to-foot body composition analyzer (Tanita Cooperation, Tokyo, Japan) was used to assess bioelectrical impedance. Measurements were collected at 50 Hz using the standard setting after manually inputting the measured gender, age, and height of the patient.

### 2.2. Sample Collection and Laboratory Analyses

Blood samples were collected from 46 patients and were immediately placed on ice, clarified by centrifugation at 3.000 g for 15 min at 4 °C, and kept frozen at −80 °C until assayed. Serum levels of IL-6, ZAG, Nesfatin-1, Leptin, and Adiponectin were measured by ELISA enzyme immunoassays (R&D Systems, Minneapolis, MN, USA) according to the manufacturer’s protocol. In total, 100 µL of serum was used for each assay. The level of absorbance was measured on an automatic plate reader (ChroMate 4300, Awareness Technology, Inc., Palm City, FL, USA). The manufacturer’s guidelines were followed when creating standard curves. hsCRP levels were measured by immunoturbidimetry (Cobas 8000 analyzer, Roche, Switzerland). In addition, total cholesterol, HDL-C, LDL-C, and TG were determined with colorimetry (Cobas 8000, Roche, Switzerland).

### 2.3. Statistical Analysis

The Shapiro–Wilk test was used to assess the normality of the data distribution. The mean and standard deviation were calculated for numerical values from the normal distribution. Linear regression was performed to compare the relationship between BMI and markers and the relationship between the two markers. Pearson correlation was used to examine the correlation between ZAG and Leptin, ZAG and Adiponectin, IL-6 and ZAG, IL-6 and Leptin, and IL-6, lipid profile, and hsCRP. One-way analysis of variance (ANOVA) was used to determine correlations across the population. A significance level of *p* = 0.05 was adopted for all analyses. To process all the data, OriginPro 2021 version 9.8 was used (OriginLab Corporation, Northampton, MA, USA).

## 3. Results

### 3.1. Characteristics of All Patients Enrolled in the Study Divided According to BMI

Patients were divided into three groups: normal body weight (BMI: 18.50–24.99), overweight (BMI: 25.0–29.99), and obese (BMI: ≥30) according to the data contained in the WHO report [25].

**Figure 1:**
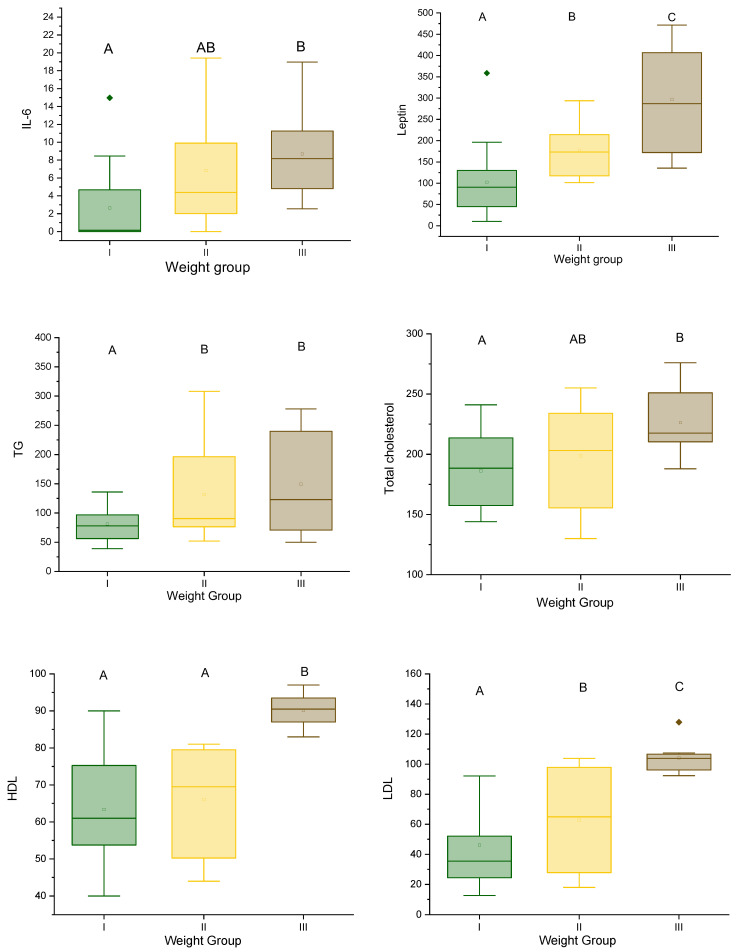
Differences in IL-6, Leptin, Adiponectin, and hsCRP levels in weight in groups with normal body weight, overweight, and obesity. Different letters (A, B, C) at the top of each box indicate statistically significant differences after one-way ANOVA with post hoc Tukey’s test (*p* < 0.05).

The mean concentration of IL-6 in the group with normal body weight was 2.64 ng/mL; in the overweight group, it was 6.83 ng/mL; and in the group with obesity, the mean concentration was the highest and amounted to 8.70 ng/mL. Overall, the average IL-6 level in the overweight group was detected to be 158.71% higher than in the normal-weight group. The average level of IL-6 in the obese group was detected to be 229.55% higher compared to the group with normal body weight (Figure 1, Table 1).

The mean concentration of ZAG in the group with normal body weight was 791.46 ng/mL; in the overweight group, it was 680.2 ng/mL (14.06% lower than in patients with normal weight); and in the group with obesity, the mean concentration was 380.65 ng/mL (56.16% lower than in the group with normal body weight).

There were no statistically significant differences between the groups for Nesfatin-1. The mean concentration of Leptin in the group with normal body weight was 102 ng/mL; in the overweight group, it was 175.7 ng/mL; and in the group with obesity, the mean concentration was 296.95 ng/mL. Overall, the average Leptin levels in the overweight group were detected to be 41.95% higher than in patients with normal weight. The average Leptin levels in obese patients were detected to be 65.65% higher than in the group with normal body weight (Figure 1).

The mean concentration of Adiponectin in the group with normal body weight was 36.5 μg/mL; in the overweight group, it was 28.87 μg/mL; and in the group with obesity, the mean concentration was 39.675 μg/mL (Figure 1). 

The highest level of hsCRP was found in overweight patients, with the average concentration being 2.5 mg/L—which was 54.4% higher than the group with normal body weight and lower than the obese group by 50.59%. 

The lipid profile showed statistically significant changes in all parameters (total cholesterol, triglycerides, LDL-C, and HDL-C) with increasing body weight. The results are presented in Table 1 and Figure 1.

### 3.2. The Correlations between the IL-6, ZAG, Nesfatin-1, Leptin, Adiponectin, HDL-C, LDL-C, TG and HsCRP levels and BMI in Patients with Different BMIs (Normal, Overweight, Obese)

**Figure 2:**
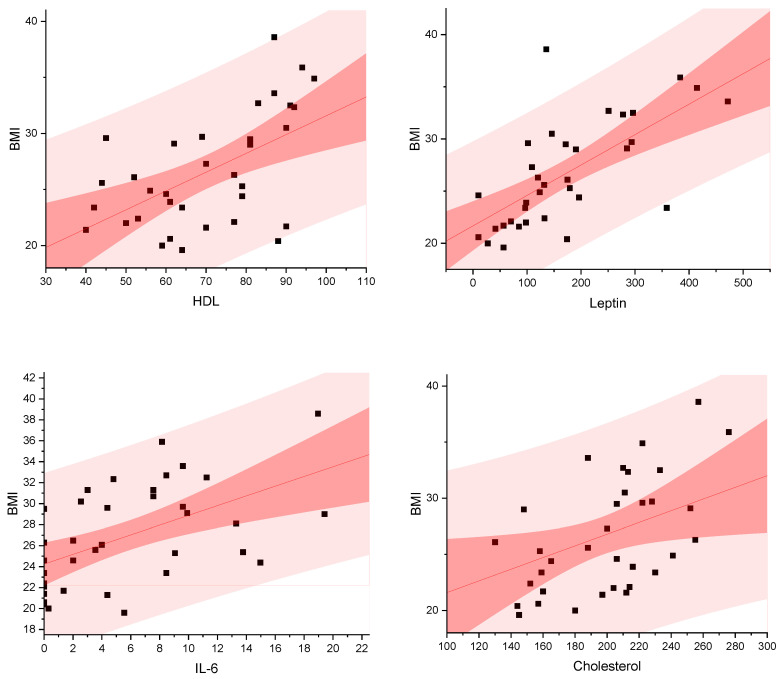

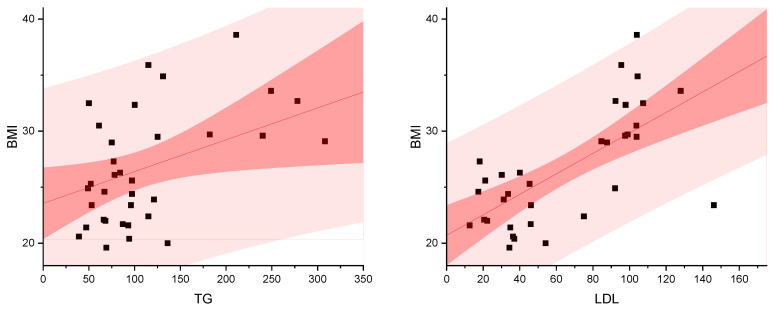
Correlations between the amount of body fat represented by the BMI equation (kg/m^2^) and IL-6 (pg/mL), Leptin (ng/mL), total cholesterol [mg/dL], HDL-C [mg/dL], LDL-C [mg/dL], and TG [mg/dL].

The correlation analysis reveals a statistically significant, upward trend for IL-6 (*p* = 0.0008) and Leptin (*p* = 0.00001). A similar tendency was seen for hsCRP, but without statistical significance (*p* = 0.113), wherein an increase in the level of these markers correlated with a concurrent increase in the amount of fat tissue (presented using the BMI equation). For other tested markers, the correlation analysis showed no statistically significant differences (Adiponectin *p* = 0.578, ZAG *p* = 0.241, Nasfatin-1 *p* = 0.858). The results are presented in Figure 2:

### 3.3. The Correlations between the Concentration of IL-6 and Concentration of ZAG, Leptin, Total Cholesterol, HDL-C, LDL-C, TG, and hsCRP for Patients with Normal Body Mass, with Overweight, and with Obesity (All Patients without Division into Groups)

Between IL-6 and Leptin concentrations, a positive average correlation is observed for patients with normal body mass, overweight, and obesity (Pearson: 0.51, *p* = 0.006). As the concentration of IL-6 rises, there is a corresponding increase in the concentration of Leptin for patients with normal body mass, overweight, and obesity.

**Figure 3:**
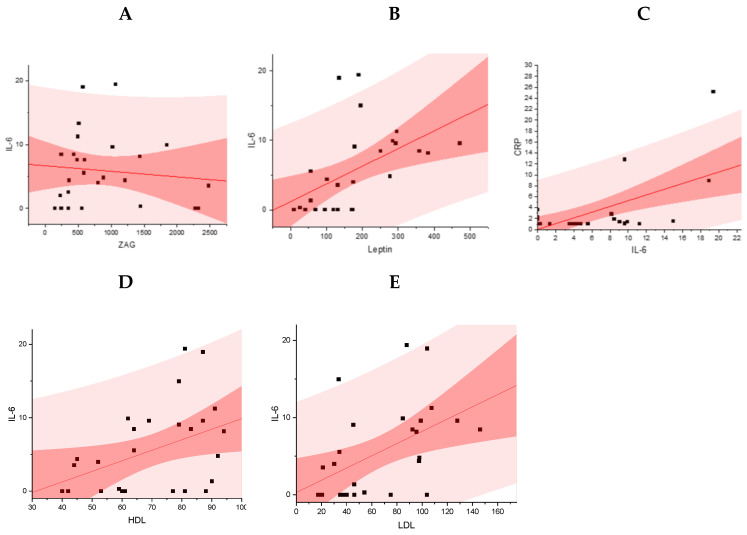
Association between IL-6 [pg/mL] and ZAG [ng/mL] (**A**), Leptin [ng/mL] (**B**), HsCRP [mg/L] (**C**), HDL-C [mg/dL] (**D**), and LDL-C [mg/dL] (**E**).

A weak positive correlation is observed between the concentrations of IL-6 and hsCRP for patients with normal body mass, overweight, and obesity (Pearson: 0.6, *p* = 0.0009). An increase in the concentration of IL-6 correlates with an increase in the concentration of hsCRP. A strong and positive correlation was observed between IL-6 and HDL-C and LDL-C (Pearson: 0.4, *p* = 0.009, and Pearson: 0.49, *p* = 0.003, for LDL-C and HDL-C, respectively). A non-significant correlation was found for IL-6 and the level of triglycerides (*p* > 0.05). The results are presented in Figure 3A–E:

### 3.4. The Correlations between the Concentration of ZAG and the Concentration of HDL-C and LDL-C for Patients with Normal Body Mass, Overweight, and Obesity (All Patients without Division into Groups)

**Figure 4:**
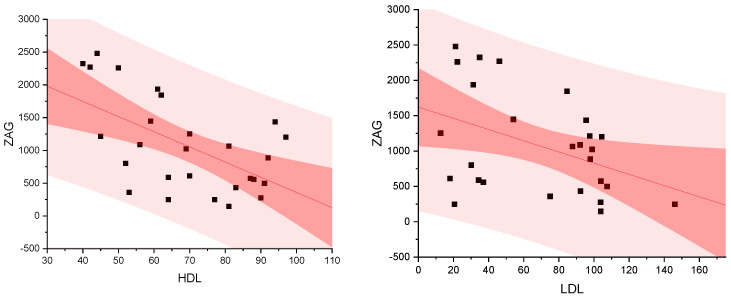
Association between ZAG [pg/mL], LDL-C [mg/dL], and HDL-C [mg/dL].

Between ZAG, Leptin, and Adiponectin, a negative association, but no correlation, was observed for patients with normal BMI, overweight, and obesity. The levels of ZAG protein were significantly negatively correlated with the levels of HDL-C and LDL-C cholesterol (Pearson: −0.57, *p* = 0.001, and Pearson −0.41, *p* = 0.029, for HDL-C and LDL-C, respectively). No statistical differences were found for the relationship between ZAG levels and levels of triglycerides. The results are presented in Figure 4:

## 4. Discussion

The key result of our research is increased levels of IL-6 and Leptin in patients with abnormal body weight. Additionally, we observed that patients with normal body weight (BMI: 18.50–24.99) had more than two times higher levels of ZAG than obese patients (BMI ≥ 30). Chinese scientists investigated this correlation in patients with metabolic syndrome (MetS) [26]. They showed that patients with MetS and central obesity had reduced levels of ZAG. However, they obtained a significant correlation. Another study by these scientists was conducted on mice. For 8 weeks, they injected a ZAG expression plasmid (5 µg/injection, four times a week) in HFD-induced obese mice. It found that overexpression of ZAG lowered the body weight and increased the glucose tolerance of obese mice [16]. Also, another group of scientists performed a study on obese patients who underwent two different weight loss interventions: Roux-En-Y Gastric Bypass (RYGB) surgery or a very-low-calorie diet (VLCD). The study showed a significant reduction in ZAG for patients in the RYGB group but not in the VLCD group. The results were likely because patients undergoing RYGB lost more body fat than those undergoing VLCD [27].

In our study, patients with obesity had 1.2 times higher concentrations of Adiponectin than those with normal body weight. However, no significant differences were observed. Similar results had been noticed by Antoniak-Pietrynczak et al., 2023, where no statistically significant correlations between the BMI and the concentration of FPG, HbA1c, hsCRP, TG, IL-10 and Adiponectin concentration were found in patients with overweight and obesity [21]. 

In previous studies, ZAG was shown to increase lipid oxidation by expressing the mitochondrial uncoupling protein 1 gene in brown and white adipose tissue, thereby affecting the regulation of thermogenesis [19]. It is recognized that the level of ZAG is inversely proportional to the amount of body fat [28]. Recent studies have shown that the level of ZAG was significantly lower in patients with high body fat levels compared to those with normal levels [18]. Our results showed a negative correlation between the levels of ZAG protein and the levels of HDL-C and LDL-C, suggesting that the level of ZAG is also inversely proportional to the amount of cholesterol. 

The increase in the volume of fat cells affects the release of Leptin, which, by binding to receptors in the brain (in the hypothalamus) [11], reduces the amount of food intake and increases calorie burning. Leptin affects the appetite through the Central Nervous System (induction of anorexic mechanisms). It acts as a negative feedback mechanism between adipose tissue and the hypothalamus: the growth of fat cells leads to an increase in Leptin levels, suppressing appetite. Leptin also affects the proliferation and inhibition of beta cell apoptosis [7]. However, a positive energy balance of food eaten for a long time causes changes in Leptin expression, leading to the formation of Leptin resistance [12]. Obese patients may also have a mutation in the Leptin gene, which produces a non-functional protein, and in the gene encoding the Leptin receptor—the Leptin signal is not received by the brain [11]. Our results showed a positive correlation between the concentration of Leptin and the amount of body fat. Patients with normal body weight had almost three times lower levels of Leptin than obese patients. Other studies confirm our results. In one of them, studies were conducted on 122 overweight and obese adolescents with type 1 diabetes. The study confirmed the correlation between increased body fat levels and higher Leptin levels [29,30,31].

Overall, current research emphasizes that increasing Leptin levels leads to the development of resistance to it. Hyperleptinemia is one of the components leading to obesity. Additionally, cytokines play a role in the development of inflammatory states. IL-6 is transiently produced during infection and tissue damage, stimulating acute-phase responses, hematopoiesis, and immune responses. Dysregulated continuous cytokine synthesis can lead to chronic inflammation and many chronic diseases [32]. Chronic inflammation occurs in overweight and obese patients, with their IL-6 levels being higher than in healthy patients. The mean IL-6 concentration was more than 2.5 times higher in overweight patients and more than 3 times higher in obese patients compared to patients with a normal BMI. Based on our results, IL-6 can be assessed as a marker in managing obesity. The results were obtained by a team that took biopsies from patients with different BMIs, then determined IL-6 levels and confirmed by immunohistochemistry, RT-PCR, and confocal microscopy [33]. Higher levels of IL-6 in obese patients have also been investigated in other studies [34,35]. Increased levels of IL-6 associated with inflammation greatly affect the secretion of hsCRP from the liver and its expression and secretion from adipose tissue. Additionally, there is a correlation between a decrease in Adiponectin and an increased level of hsCRP [36]. In ongoing inflammation, C-reactive protein is produced by hepatocytes. Obesity affects hsCRP levels with macrophages, activating inflammatory signals (like cytokines) that further stimulate the liver to release large amounts of hsCRP. Elevated hsCRP levels increase the risk of developing cardiovascular diseases and atherosclerosis by activating the complement system, damaging tissues, or activating endothelial cells [37].

An increase in Nesfatin-1 in areas of the brain regulates food intake. It also affects the emptying of the stomach and its contractility. Reported effects also include thermoregulation and the cardiovascular system (such as increased blood pressure) and activation of sympathetic nerves [13]. Nesfatin-1, like Leptin, suppresses the appetite by inhibiting anorexigenic neurons [14]. The probable mechanism of appetite suppression with Nesfatin-1 was studied by injecting it into various places in the brain. Consequently, food intake is reduced by activating the Leptin-independent melatonin system. In other studies, this has been explained as the inhibition of the orexigenic substance by Nesfatin-1 or as an inhibitory neurotransmitter regulating gastric motility [15]. In our work, we also studied Nesfatin-1 and hsCRP. However, our results failed to show a statistically significant relationship between BMI and the level of these biomarkers, which could be due to the patients’ young age and lack of co-morbidities associated with obesity. Other publications show a link between obesity, Nesfatin, and chronic complications [13,14,15].

Adipokines, which are satiety hormones, are partly associated with adipose tissue through their partial production by adipocytes [36]. Therefore, their synthesis is influenced by the size of adipocytes. In addition, obesity causes reduced sensitivity to their level—the patient does not feel full despite eating food [21]. Therefore, due to the inability to ensure satiety, an obese person consumes more calories, and this negatively affects further weight gain [37]. In this case, introducing a reduction in calorie intake is very difficult for this group of patients—they constantly feel hungry, which affects their level of satisfaction or well-being. Consequently, it is harder for them to function in the community. From this, it follows that obesity, in addition to health effects, also affects social life [22]. 

Serum measurements can be controlled for adipokine levels during weight loss to verify that the effect of lowering BMI will not be temporary but sustained beyond the end of the treatment [17]. Due to the relationship between the concentration of inflammatory markers, most adipokines, and the amount of adipose tissue in overweight and obese patients, these parameters can serve as biomarkers for monitoring interventions and assessing patient conditions [21]. The measurements can also control the effectiveness of introducing an intervention aimed at reducing the amount of excessive fat tissue by measuring “hunger hormones” and thus checking whether the patient will feel full faster than before the introduction of the given intervention, which means they will consume a smaller portion of food (calories) during one meal [26].

Obesity causes chronic, systemic inflammation, which is correlated with inflammatory markers (as in our study—IL-6). This is due to the accumulation and activation of pro-inflammatory macrophages in metabolic tissues. In addition, other types of immune cells are engaged to bring the body back into homeostasis. The occurring inflammation influences the development of diseases associated with obesity—including insulin resistance, osteoarthritis, and dysfunction of beta cells, leading to type 2 diabetes. The chronicity of this condition also contributes to complications related to diabetes (including Alzheimer’s) or liver cell dysfunction (leading to non-alcoholic fatty liver disease). Cardiovascular diseases, retinopathy, and nephropathy also often develop [38].

Due to the large number of possible disorders, it is important to control markers of inflammation in obesity, including the tested IL-6 to react as quickly as possible. This can be achieved, for example, through treatment [36]. In addition, controlling selected inflammatory markers can also be used in patients at risk of obesity. This helps in implementing prophylactic intervention against obesity as soon as possible but also to make these patients aware of the risk of obesity. Blood tests can also be utilized to control the effectiveness of the intervention in groups of patients with incorrect BMI [21,22,23].

## 5. Conclusions

Excess body weight can be characterized by chronic inflammation. Our results show that as body weight increases, IL-6 levels increase, indicative of inflammation. Positive associations of increased IL-6 levels with obesity in relation to levels in the case of normal body weight suggest a correlation with the ongoing inflammation in obese people, increasing the risk of developing various diseases in the future. Additionally, based on our research, we can conclude that the increasing level of body weight, and therefore fat tissue mass, is associated with increased levels of Leptin. Increasing Leptin levels correlating with increasing body weight suggests a greater need for food intake, which leads to further weight gain. Furthermore, we noticed a relationship between the levels of Il-6 and Leptin, which may be related to the correlation of excessive appetite (and food intake) with the occurring inflammation. Based on our findings, we hypothesize that IL-6 and Leptin can serve as biomarkers for monitoring the interventions used and assessing the patient’s condition during these interventions. 

## Figures and Tables

**Table 1:** Characteristics of patients depending on BMI.

BMI	18.5–24.99	25–29.99	≥30	*p*
Age (years)	28 ± 4	27 ± 5	28 ± 5	0.729
IL-6 (pg/mL)	2.64 ± 4.41	6.83 ± 6.02	8.70 ± 3.01	0.002 *
ZAG (pg/mL)	1154.5±791.46	930.54±680.20	675.21 ± 380.65	0.232
Nesfatin-1 (pg/mL)	2.82±9.76	6.01±13.37	0.00 ± 0.00	0.378
Leptin (ng/mL)	102.06 ± 86.79	175.7 ± 67.58	296.95 ± 121.47	0.0001 *
Adiponectin (μg/mL)	36.68±19.69	28.87±11.08	39.68 ± 26.22	0.469
hsCRP (mg/L)	1.14±0.32	5.06±7.95	2.50 ± 2.66	0.111
Total cholesterol (mg/dL)	186.37 ± 32.13	198.70 ± 42.85	226.25 ± 28.33	0.042 *
TG (mg/dL)	81.06 ± 28.37	131.8 ± 84.52	149.37 ± 86.11	0.036 *
HDL-C (mg/dL)	63.37 ± 14.55	66.20 ± 14.53	90.12 ± 4.42	0.012 *
LDL-C (mg/dL)	46.18 ± 33.66	62.70 ± 34.84	104.11 ± 10.89	0.005 *

* The value of *p* < 0.05 was regarded as statistically significant. Data are presented as mean ± standard deviation. The *p*-value was calculated based on all patient groups to show the difference between the concentrations of IL-6, ZAG, Nesfatin-1, Leptin, Adiponectin, hsCRP, total cholesterol, TG, HDL-C, and LDL-C in the whole group of examined patients with different BMIs. Abbreviations: BMI, body mass index; IL-6, Interleukin-6; ZAG, Zinc-α2-glycoprotein; hsCRP, high-sensitivity C-reactive protein; TG, triglycerides; HDL-C, high-density lipoprotein cholesterol; LDL-C, low-density lipoprotein cholesterol.

## Data Availability

The data presented in this study are available on request from the coresponding author. The data are not publicly available due to privacy.
